# Diffusion MRI in the cortex of the brain: Reducing partial volume effects from CSF and white matter in the mean diffusivity using high *b*‐values and spherical b‐tensor encoding

**DOI:** 10.1002/mrm.30552

**Published:** 2025-06-04

**Authors:** Cornelia Säll, Nicola Spotorno, Pia C. Sundgren, Danielle van Westen, Carl‐Fredrik Westin, Filip Szczepankiewicz, Markus Nilsson

**Affiliations:** ^1^ Department of Medical Radiation Physics Lund University Lund Sweden; ^2^ Clinical Memory Research Unit, Department of Clinical Sciences Malmö Lund University Lund Sweden; ^3^ Institution for Clinical Sciences, Lund, Diagnostic Radiology, Lund University Lund Sweden; ^4^ Image and Function, Skåne University Hospital Lund Sweden; ^5^ Lund University Bioimaging Center, Lund University Lund Sweden; ^6^ Department of Radiology Brigham and Women's Hospital, Harvard Medical School Boston Massachusetts USA

**Keywords:** cortex, diffusion MRI, high *b*‐values, mean diffusivity, partial volume effects, spherical b‐tensor encoding

## Abstract

**Purpose:**

The mean diffusivity (MD) is sensitive to the microstructure of the cortex. However, partial volume effects with CSF and white matter (WM) may obscure pathology‐related alterations. This work investigates both existing approaches and a novel approach for reducing partial volume effects.

**Theory and Methods:**

A bias in MD arises due to partial volume effects, higher‐order terms, and the noise floor in magnitude data.

We propose to reduce this bias by using high *b*‐value encoding to limit partial volume effects with CSF, spherical b‐tensor encoding to reduce the influence of higher‐order terms, and super‐resolution acquisition and reconstruction to suppress the noise floor. This approach was investigated, along with established approaches (inversion recovery and free water elimination) for reducing partial volume effects, using simulations and in vivo data.

**Results:**

High *b*‐value diffusion MRI with spherical b‐tensor encoding reduced partial volume effects with CSF relative to conventional diffusion MRI. Maximum errors decreased from 0.51 to 0.01 μm^2^/ms in simulations. In vivo, the median absolute deviation of cortical MD decreased from 0.17 to 0.06 μm^2^/ms, whereas the median decreased slightly from 0.77 to 0.73 μm^2^/ms. The other methods yielded bias from either CSF, WM, or model assumptions.

**Conclusion:**

The mean diffusivity of the cortex can be mapped in high precision with reduced influence of partial volume effects with CSF and WM matter using high *b*‐values and spherical b‐tensor encoding and super‐resolution reconstruction.

## 1 INTRODUCTION

Diffusion MRI (dMRI) can detect microstructural changes in the cortex in neurological conditions. For example, elevated cortical mean diffusivity (MD) has been found in patients with Alzheimer's disease,^1^, ^2^, ^3^ multiple sclerosis,^4^ and epilepsy.^5^ Cortical MD has also been shown to track changes along the Alzheimer's disease continuum.^3^, ^6^, ^7^ However, accurate and precise mapping of cortical MD remains challenging due to partial volume effects (PVE), which arise when the imaging voxels are larger than the investigated anatomical structures. A typical dMRI protocol has an isotropic resolution of 2 mm, whereas the cortical ribbon is between 1 and 4.5 mm thick.^8^ This yields PVE from CSF, which inflates MD estimates. This is problematic because the bias increases with decreasing cortical thickness,^9^ and PVE may thus confound MD measures in conditions associated with cortical thinning such as Alzheimer's disease^10^ and mild cognitive impairment.^11^ Cortical MD can also be biased by PVE with subcortical white matter (WM).^12^ A method for limiting the PVE of both CSF and WM is thus warranted.

Approaches for limiting PVE have been proposed. One is to simply increase the spatial resolution so that a larger proportion of voxels contain only gray matter (GM).^13^, ^14^ However, this reduces SNR in proportion to the relative voxel volume. SNR can be restored by averaging at the expense of scan time, with the number of averages given by the inverse square of the relative volume.^15^ For example, reducing voxel sizes from 1.5 to 1.0 mm isotropic with preserved SNR thus requires an almost sixfold increase in scan time, highlighting the tradeoff between resolution, imaging time, and SNR. This is reflected in the scan times used in high‐resolution DTI studies. At resolutions above 2 mm, diffusion weighted images can be obtained in a fast fashion. For example, Morris et al. collected 2.2 mm isotropic whole‐brain data in about 1 min.^16^ Resolutions around 1.5 mm are obtainable with longer but clinically feasible scan times: Horne et al. obtained 1.8 × 1.8 × 1.7 mm^3^ whole‐brain data in 6:30 min,^17^ and Bag et al. obtained 1.8 mm isotropic data in 4:20 min^18^ (both approximated from presented parameters), whereas Little et al. obtained 1.5 mm isotropic whole brain data with this resolution in as little as 3:30 min.^19^ However, even at a 1.5 mm resolution, Little et al. reported a poor repeatability of MD values, hypothesized to stem from PVE. Investigations of PVE at even higher resolutions have yielded mixed results. For example, Mournet et al. found no improvement PVE‐wise when comparing 1.5 and 1.3 mm isotropic resolution data,^20^ while Merenstein et al. observed reduced PVE from WM when comparing 1.5 and 1.0 mm isotropic resolution^12^ and McNab et al. found PVE from both WM and CSF in the outer and inner layers of the cortex in 1 mm isotropic resolution in vivo data.^21^ Although submillimeter resolutions are achievable, previous studies show that this demands longer scan times: 20 min for 1.0 mm isotropic data,^22^ 96 min for 0.85 mm isotropic data,^23^ and 80 min for 0.5 mm isotropic data.^24^ Although the relationship between voxel volume and scan time does not strictly follow the rule indicated above, the relationship between resolution and scan time is clear. At extremely high resolutions, the impact of PVE is expected to be limited, but scan times become intractable for clinical use and typically also demand non–off‐the‐shelf hardware and sequences. Thus, while high‐resolution acquisition reduces PVE, alternative approaches may be more effective and deserve additional attention.

Another approach is to null the CSF signal using fluid‐attenuated inversion recovery (FLAIR). This has a large impact on estimated cortical MD. For example, Falconer and Narayana^25^ reported that it decreased cortical MD from 1.5 to 0.9 μm^2^/ms when comparing conventional DWI to FLAIR‐DWI. However, both scan time and SNR are negatively affected by FLAIR‐DWI. For example, Ma et al.^26^ reported a 20% SNR reduction in GM with FLAIR‐DWI compared to conventional DWI, along with an increase in scan time from 3:30 to 8:25 min. Additionally, effective fluid suppression is challenging. For example, the T_1_ of CSF varies with body temperature,^27^ variations in the B_1_‐field affect the flip angle,^28^, ^29^ and spin history effects caused by cross‐talk between slices affect T_1_.^30^ Although adiabatic pulses reduce the effects of B_1_‐inhomogeneities,^31^ and slice gaps reduce cross‐talk, these factors complicate the choice of TI and may cause incomplete fluid suppression.^25^ A related approach is to use short repetition times.^32^ Here, the long T_1_ of CSF yields incomplete saturation recovery and, thus, signal suppression. However, this does not fully eliminate the CSF signal and reduces tissue SNR. Yet another approach is to use diffusion encoding for fluid suppression. Here, the rapid signal attenuation associated with the high diffusivity in CSF is used for suppression. Salminen et al. proposed using a pair of higher *b*‐values for free‐water suppressed MD mapping^33^ (e.g., 0.6 and 1.5 ms/μm^2^, compared to the conventional 0 and 1 ms/μm^2^).

Alternatively, postprocessing methods can be used to correct PVE. Here, the signal in a voxel is assumed to be the sum of that from tissue and free water. The relative weights of these are then estimated using model fitting. An example is the free water elimination (FWE) method,^34^ which has been implemented to estimate MD^35^ and mean kurtosis (MK).^36^, ^37^ These methods can be applied to data already collected with conventional methods, thereby circumventing the drawbacks discussed above. However, compartment models are known to yield a degenerate inverse problem, particularly for single‐shell data. This makes the solutions nonunique and, therefore, not robust^38^, ^39^ and dependent on regularizations^38^, ^39^, ^40^ or assumptions.^41^ Such procedures may introduce additional bias.

In this paper, we propose a new approach for reducing PVE when measuring cortical MD. The approach uses diffusion‐based fluid attenuation with a relatively high minimum *b*‐value (1.5 ms/μm^2^) to fully suppress CSF in combination with spherical b‐tensor encoding (STE) instead of linear b‐tensor encoding (LTE) to reduce PVE from WM. Although this approach reduces PVE, it comes at the expense of low SNR due to the high *b*‐value encoding and the relatively long TEs associated with STE. To partially address this problem, we use super‐resolution acquisition and reconstruction to minimize bias from the rectified noise floor.^42^

This paper aims to characterize technical aspects, contrast properties and possible limitations of the proposed approach, and characterize other methods for suppressing CSF, namely FLAIR‐DWI and FWE. This is done using simulations and in vivo measurements.

## 2 THEORY

Assuming a distribution of Gaussian non‐exchanging environments, the dMRI signal can be expressed based on the cumulant expansion as^43^, ^44^, ^45^:

(1) $$S=S_{0}\exp\left(-B:\left\langle D\right\rangle +\frac{1}{2}B^{⨂2}:\mathbb{C}\right),$$

where **B** is the diffusion encoding b‐tensor, ⟨**D**⟩ is the voxel‐averaged diffusion tensor, “:” denotes the tensorial inner product, **B** ^⨂2^ denotes the outer product of the b‐tensor with itself, and ℂ denotes the diffusion‐tensor covariance.

Averaging the signal across many rotations of the b‐tensor (powder averaging)^46^ yields a rotationally invariant signal, given by^45^, ^47^, ^48^

(2) $$S=S_{0}\exp\left(-b\cdot \mathrm{MD}+\frac{1}{6}b^{2}MD^{2}\left(MK_{I}+b_{∆}^{2}MK_{A}\right)\right),$$

where MK_I_ is the isotropic kurtosis, MK_A_ is the anisotropic kurtosis, and *b*_Δ_ reflects the b‐tensor shape, which is 1 for conventional DWI (LTE) and 0 for STE.^49^ Notably, MK_A_ = 0 in environments with pure isotropic diffusion, and MK_I_ = 0 in environments with a single diffusivity.^48^

### 2.1 Sources of bias when estimating MD

Our goal is to estimate the cortical MD, unconfounded by PVE with CSF and WM. The MD is computed from powder‐averaged signals acquired with two *b*‐values (*b*_1_ and *b*_2_), as:

(3) $$\mathrm{MD}=\frac{\log\left(S\left(b_{2}\right)\right)-\log\left(S\left(b_{1}\right)\right)}{b_{2}-b_{1}}.$$

The estimated MD may, however, deviate from the actual MD in tissue (here denoted MD_t_), such that MD = MD_t_ + *ϵ*. Here, we analyze two sources of the bias (ϵ): higher‐order terms and partial volume effects.

### 2.2 Higher‐order terms

The diffusional kurtosis yields a bias, given by

(4) $$\epsilon =\frac{1}{6}\frac{\left(b_{1}^{2}-b_{2}^{2}\right)}{b_{1}-b_{2}}\left(MK_{I}+b_{∆}^{2}MK_{A}\right){\mathrm{MD}}^{2}.$$

In conventional DWI, both MK_I_ and MK_A_ contribute to the bias, whereas for STE only MK_I_ contributes.^48^ The similarity between GM and WM thus increases with STE because MK_I_ is more similar between the tissues than MK_A_.^47^

### 2.3 Partial volume effects

The presence of PVE yields a bias in MD due to contamination from other signal types. We analyze this by expressing the signal as a weighted sum according to

(5) $$S_{\mathrm{tot}}\left(b\right)=\sum_{i=1}^{n}f_{i}S_{i}\left(b\right),$$

where *f*_i_ is the signal fraction and $S_{i}\left(b\right)$ the diffusion‐weighted signal from the tissue i. The error in MD when two tissues are present in a voxel can be expressed as

(6) $$\epsilon =\log\left(1+\frac{f_{c}S_{c}\left(b_{2}\right)}{\left(1-f_{c}\right)S_{t}\left(b_{2}\right)}\right)-\log\left(1+\frac{f_{c}S_{c}\left(b_{1}\right)}{\left(1-f_{c}\right)S_{t}\left(b_{1}\right)}\right),$$

where the contaminating tissue is labeled ‘c’, and the tissue of interest is labeled ‘t’. We simplify this expression by defining

(7) $$g\left(b\right)=\frac{S_{c}\left(b\right)}{S_{t}\left(b\right)},$$

and approximating the logarithms by their first‐order Taylor expansion, which yields

(8) $$\epsilon \approx \frac{f_{c}}{1-f_{c}}\left(g\left(b_{2}\right)-g\left(b_{1}\right)\right).$$

Equation (8) shows that low errors can be obtained by three strategies: reducing the fraction of the contaminating signal so that *f*_c_ approaches zero; attenuating the contaminating signal so that both *g*(*b*_1_) and *g*(*b*_2_) approach zero; and making the signal‐versus‐b curves from the two tissues as similar as possible such that *g*(*b*_1_) and *g*(*b*_2_) approach 1, meaning that the two terms cancel. Strategy 1 can be achieved by increasing the resolution and strategy 2 by inversion recovery or high *b*‐value encoding, if the contaminating signal has a higher diffusivity than the tissue of interest. Strategy 3 is relevant for minimizing the difference between GM and WM, for example, using STE instead of LTE.

## 3 METHODS

In this study, simulations and in vivo data analysis were performed to investigate the impact of PVE from CSF and WM on MD estimation in GM. The simulations studied MD accuracy and precision along with effect size across signal contaminations and noise iterations, while the in vivo data was analyzed by extracting the median and quartiles of cortical MD values along with their median absolute deviation (MAD). These analyses were performed across a set of approaches, with imaging parameters approximately matched between the simulations and in vivo data.

### 3.1 Simulations

Single‐voxel simulations were performed to investigate how MD is affected by PVE with CSF and WM. Three simulations were performed: analysis of MD accuracy at different levels of CSF and WM contamination, analysis of effects on MD accuracy and precision from PVE with CSF and WM as well as variations in T_1_ of CSF, and analysis of expected effect sizes in group comparisons between healthy and non‐healthy GM. Three acquisition techniques were evaluated: DWI with LTE (DWI‐LTE), DWI with FLAIR and LTE (DWI‐FLAIR), and DWI with STE (DWI‐STE) at different *b*‐value combinations. FWE was not included in these simulations because it relies on spatial regularization.

All simulations assumed a powder‐averaged signal, computed as the sum of that from GM, CSF, and WM, which were obtained from Equation 2 with effects of relaxation included using

(9) S0=PD1−exp−TRT1exp−TET2,

for sequences without inversion recovery and

(10) S0=PD1−2·exp−TIT1+exp−TRT1exp−TET2,

for sequences with inversion recovery. Here, PD is the proton density, TE is the echo time, TR is the repetition time, and TI is the inversion time. In the simulation, these parameters were chosen to match the protocol parameters used in vivo. The tissue‐specific parameters (see Table 1) were chosen based on 3 T studies of T_2_,^50^, ^51^ T_1_,^52^, ^53^ diffusional kurtosis,^40^ MD,^54^ and GM abnormalities.^1^

**TABLE 1:** Parameters used for each tissue compartment in the single‐voxel simulations.

	CSF	WM	GM	Lesion
PD	1.0	0.7	0.8	0.8
*T*_1_ (ms)	4500	800	1600	1700
*T*_2_ (ms)	1500	80	100	100
MD (μm^2^/ms)	3.00	0.75	0.75	1.50
MK_A_	0.00	0.82	0.43	0.00
MK_I_	0.00	0.27	0.34	0.00

Abbreviations: GM, gray matter; MD, mean diffusivity; MK, mean kurtosis; PD, proton density; WM, white matter.

#### 3.1.1 Part 1

Signal values were extracted for the three acquisition techniques at low *b*‐values (LTE_low_, FLAIR_low_, and STE_low_; *b* = 0 and 1 ms/μm^2^) and high *b*‐values (LTE_high_, FLAIR_high_, and STE_high_; *b* = 1.5 and 2.5 ms/μm^2^). The full range of CSF and WM contamination was studied (0–100%) over 10^6^ noise iterations.

#### 3.1.2 Part 2

PVE from CSF and WM were simulated with tissue fractions between 0% and 30% to mimic contamination fractions from a previous study,^55^ T_1_‐variations between 4.5 and 4.6 s were used to mimic the effects of temperature fluctuations between 36 and 37.8°C.^56^, ^57^ Simulations were performed for 1000 Rice‐distributed noise realizations with SNR = 200 at TE = 0 ms, *b* = 0 ms/μm^2^, and TR = ∞ ms in GM, corresponding to shot‐wise SNRs in GM between 40 and 125 in our protocols and *b*‐value combinations. For all protocols, signal value pairs were calculated using b_1_ = 0–2 ms/μm^2^, *b*_2_ = *b*_1_ + 0.2–2.2 ms/μm^2^, and *b*_Δ_ = 1 for DWI‐LTE and DWI‐FLAIR but b_Δ_ = 0 for DWI‐STE. Higher maximal *b*‐values demand longer TEs, which reduce the SNR. This was accounted for by adjusting the TEs according to:

(11) $$\mathrm{TE}=t_{\text{static}}+t_{\mathrm{ref}}{\left(\frac{b_{2}}{b_{\mathrm{ref}}}\right)}^{\frac{1}{3}}.$$

Here, *t*_static_ = 30 ms is the part of the TE not related to diffusion encoding, that is half of the 90° pulse, the 180° pulse, and half the EPI‐readout time. The diffusion encoding time and *b*‐value used in vivo were represented by *t*_ref_ and *b*_ref_, respectively. This yielded TE = 45–70 ms for DWI‐LTE, 50–86 ms DWI‐FLAIR, and 65–125 ms for DWI‐STE. Additionally, the number of averages per *b*‐value were chosen to match in vivo protocols. This means that fewer averages were used for methods demanding longer repetition times (e.g., DWI‐FLAIR). Following MD estimation using Equation (3), the accuracy of the obtained values was investigated by evaluating the maximum error compared to the MD in a noncontaminated GM voxel across all simulations of different contamination levels. This metric was chosen to seek a method that yields accurate estimates in all parts of the cortex. This is desirable because low maximum errors indicate that an approach can produce MD values with low bias in all regions and individuals. Precision was evaluated using the SD between the noise iterations. Lastly, the root‐mean‐square error (RMSE) across all different contamination simulations was analyzed because it reflects both accuracy and precision.

#### 3.1.3 Part 3

Potential effect sizes when comparing healthy and abnormal GM were investigated, assuming healthy GM voxels have 0–10% WM and 0–30% CSF contamination. Abnormal GM was divided into two subgroups representing (A) a voxel‐wide increase in MD of GM by 0.1 μm^2^/ms (13%), and (B) cortical thinning simulated by replacing 10% of the GM with CSF.

Signals were generated for the four in vivo protocols. For all sequences apart from FLAIR, imaging parameters were identical to those presented in Table 2. For FLAIR, two situations were simulated: one with a perfectly timed inversion pulse, and one with an inversion pulse offset by 0.3 s. Effect sizes, in terms of Cohen's *d*, between the healthy and abnormal groups were evaluated as:

(12) $$d=\frac{\left({\bar{\mathrm{MD}}}_{\text{healthy}}-{\bar{\mathrm{MD}}}_{\text{abnormal}}\right)}{s},$$

where MD_healthy_ and MD_abnormal_ are the mean MD values of the healthy and abnormal group, respectively, and *s* is the pooled SD for both groups, as given by:

(13) $$s=\sqrt{\frac{\left(n_{\text{healthy}}-1\right)\cdot \mathrm{std}{\left(MD_{\text{healthy}}\right)}^{2}+\left(n_{\text{abnormal}}-1\right)\cdot \mathrm{std}{\left(MD_{\text{abnormal}}\right)}^{2}}{\left(n_{\text{healthy}}+n_{\text{abnormal}}-2\right)}},$$

where *n*_healthy_, and *n*_abnormal_ are the number of subjects in each group.

**TABLE 2:** Imaging parameters used for the collection of in vivo data.

	LTE_low_	FLAIR_low_*	LTE_high_	FWE_low_	STE_high_*
TE (ms)	54	65	73	73	110
TR (ms)	8300	7100	3500	3500	2100
TI (ms)	–	1800	–	–	–
*b*‐values (ms/μm^2^)	0, 1	0, 1	1, 2.5	0, 0.1, 1	1.5, 2.5
*b*_Δ_‐values	0, 1	0, 1	1, 1	1, 1, 1	0, 0
No. directions/*b*‐value	6, 30	1, 10	32, 64	2, 6, 32	16, 24
Acquired voxel‐size (mm^3^)	1.7 × 1.7 × 1.7	1.7 × 1.7 × 6.0	2.0 × 2.0 × 2.0	2.0 × 2.0 × 2.0	1.7 × 1.7 × 6.0
Reconstructed voxel‐size (mm^3^)	–	1.7 × 1.7 × 1.7	–	–	1.7 × 1.7 × 1.7
No. slices	90	26	62	62	26
FOV (mm^2^)	221 × 221 × 153	221 × 221 × 156	220 × 220 × 124	220 × 220 × 124	221 × 221 × 156
Multi‐band acceleration factor	1	1	2	2	2
Partial Fourier	6/8	6/8	7/8	7/8	6/8
Scan time	5:24	9:54	5:36	2:40	9:36

*Note*: Sequences in which super‐resolution reconstruction was implemented are marked with *. Here, six FOV rotations were collected. The same parameters were also used in the single‐voxel simulations.

Abbreviations: FLAIR, fluid attenuated inversion recovery; FWE, free‐water elimination; LTE, linear b‐tensor encoding; STE, spherical b‐tensor encoding.

### 3.2 MRI

MRI was performed using a Siemens 3 T scanner (Magnetom Prisma, Siemens Healthineers, Forchheim, Germany) in five healthy volunteers under approval by the ethics review authority and after obtaining written and informed consent. For each participant, five data sets were collected:

- Conventional DWI sequence with LTE and low *b*‐values (*b*_1_ = 0 and *b*_2_ = 1 ms/μm^2^) with six repetitions of *b*_1_ and 30 repetitions (directions) of *b*_2_ (LTE_low_).
- DWI‐FLAIR with LTE and low *b*‐values (*b*_1_, = 0 and *b*_2_ = 1 ms/μm^2^) with one repetition of *b*_1_ and 10 repetitions (directions) of *b*_2_ (FLAIR_low_).
- High angular resolution diffusion imaging (HARDI) sequence with LTE and both low and high *b*‐values, split into two protocols:
  - high *b*‐value protocol (*b*_1_ = 1.0 and *b*_2_ = 2.5 ms/μm^2^) with 32 repetitions of *b*_1_ and 64 repetitions of *b*_2_ (LTE_high_)
  - low *b*‐value protocol (*b*_1_ = 0, *b*_2_ = 0.1, and *b*_3_ = 1.0 ms/μm^2^) with two repetitions of *b*_1_, six repetitions of *b*_2_ and 32 repetitions of *b*_3_ for FWE postprocessing (FWE_low_)
- DWI with spherical b‐tensor encoding (STE) from free gradient waveforms and high *b*‐values (*b*_1_ = 1.5 and *b*_2_ = 2.5 ms/μm^2^) with 16 repetitions of *b*_1_ and 24 repetitions of *b*_2_ (STE_high_).

Protocol details of each sequence are listed in Table 2. Variations in the protocol setup are discussed as a limitation. A prototype research sequence was used for STE_high_.^47^, ^58^, ^59^ For FLAIR_low_, a range of TIs were tested to verify that the one used yielded sufficient CSF suppression (see Figure S1). Super‐resolution acquisition and reconstruction (SRR)^42^ was applied for FLAIR_low_ and STE_high_, by collecting six stacks of thick slices (6 mm) with FOVs rotated around the phase encoding (anteroposterior) direction by 0°, 30°, 60°, 90°, 120°, and 150°. SRR is based on a collection of multiple low‐resolution (high SNR) images, rotated to facilitate the reconstruction of high‐resolution images, while maintaining high SNR and reducing the effects of the rectified noise floor.^42^ Additionally, with thicker slices, the same coverage can be obtained with fewer slices. This means that a shorter TR can be used for each image‐stack, producing a “stack‐wise acceleration.” Image data with an isotropic resolution of 1.7 mm was obtained following SRR. Additionally, a high‐resolution T_1_‐MPRAGE, with voxel size of 1 × 1 × 1 mm^3^, TR = 1900, and TE = 2.54 ms was acquired for coregistration and cortex segmentation. For four participants, a 20‐channel head and neck coil was used, whereas the fifth data set was acquired using a 64‐channel head coil.

Postprocessing included denoising by Marchenko‐Pastur principal component analysis,^60^ motion‐ and eddy‐current correction using ElastiX^61^ with extrapolated reference images,^36^ and powder averaging of the magnitude signal.^62^ Following this, SRR was performed for FLAIR_low_ and STE_high_ using an implementation^42^ accessed at https://github.com/filip‐szczepankiewicz/Vis_NIMG_2021/, with a regularization strength of λ = 0.06. Image data was smoothed using a Gaussian filter with a SD of 0.5 voxels before MD estimation. FWE was performed on the low *b*‐values of the HARDI data using an implementation accessed at https://github.com/mvgolub/FW‐DTI‐Beltrami ^38^ with regularized gradient descent fitting. Segmentation was performed using FastSurfer^63^ applied to the T_1_‐MPRAGE data, whereas WM fiber tracts were segmented using TractSeg^64^ applied to the whole HARDI data. For evaluation, MD maps were coregistered to the skull‐stripped T_1_‐MPRAGE data with a rigid transform followed by a SyN‐transform using Advanced Normalization Tools (ANTs),v 2.3.5, and thus upsampled to a 1 mm isotropic resolution. From this, MD values in the cortical ribbon (right and left hemispheres, segmented in FastSurfer, with one 1 mm voxel erosion) were extracted and analyzed by median, 25th, and 75th quantile. The erosion of the cortical ribbon mask was performed to limit the possible effects of imperfect coregistration. This approach may exclude some of the cortical voxels most susceptible to PVE but cannot be used to eliminate them fully. Furthermore, potential effects of Gaussian smoothing are expected to be reduced by erosion of the mask. The MAD of MD values was calculated in GM and WM for each method. After the analysis but before displaying the parameter maps, outlier rejection was performed by smoothing the map, identifying voxels where the unsmoothed and smoothed map values differed more than a threshold value (0.2 μm^2^/ms), and replacing voxel values in the original image with those from the smoothed image. This procedure affected between 0.4 and 3% of all voxels.

## 4 RESULTS

### 4.1 Simulations

#### 4.1.1 Part 1

**FIGURE 1:**
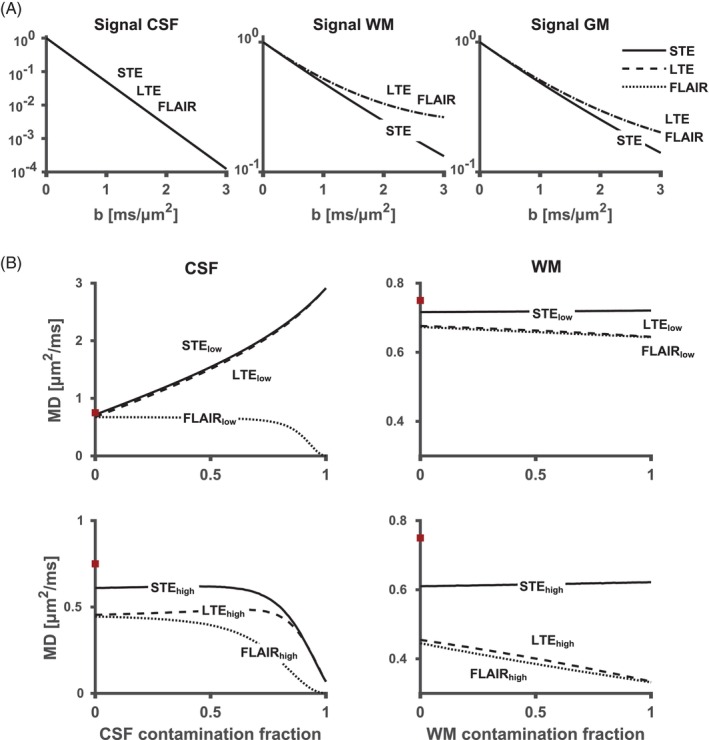
Quantification errors in MD from PVE and higher‐order signal terms. (A) From left to right: signal vs. b‐curves in pure CSF, WM, and GM. Signals without noise from conventional DWI (LTE), FLAIR (with LTE), and isotropic diffusion encoding (STE). (B) MD values in a GM voxel with different contaminations of CSF (left) and WM (right) at low *b*‐values (LTE_low_, FLAIR_low_, and STE_low_; 0 and 1 μm^2^/ms) and high *b*‐values (LTE_high_, FLAIR_high_, and STE_high_; 1.5 and 2.5 μm^2^/ms). The unbiased MD_t_ value in GM (0.75 μm^2^/ms) is marked by a red square. Altogether, the results suggest that FLAIR_low_ and STE_high_ yield the lowest errors. FLAIR, fluid attenuated inversion recovery; FWE, free‐water elimination; GM, graymatter; LTE, linear b‐tensor encoding; MD, mean diffusivity; PVE, partial volume effects; STE, spherical b‐tensor encoding; WM, white matter.

Figure 1 shows the simulated influence of PVE and higher‐order terms on the accuracy of MD. A deviation from monoexponential signal decay is observed in WM and GM. The size of this deviation depends on the diffusional kurtosis of the tissue greater for WM than GM), the b‐tensor shape (greater for LTE than STE), and the *b*‐values used (greater for higher *b*‐values). In the presence of PVE with CSF, both LTE_low_ and STE_low_ had a positive bias in MD proportional to the contamination fraction. With inversion recovery (FLAIR_low_), CSF contamination was eliminated. All techniques using high *b*‐values (LTE_high_, FLAIR_high_, and STE_high_) yielded small PVE with CSF. However, the influence of higher‐order terms increased, causing a bias of around −0.15 μm^2^/ms for STE_high_ and − 0.3 μm^2^/ms for both LTE_high_ and FLAIR_high_. The bias from PVE with WM was relatively small (generally below −0.03 μm^2^/ms) for all low *b*‐value techniques (LTE_low_, FLAIR_low_, and STE_low_) as well as for STE_high_ (at max 0.02 μm^2^/ms), whereas both LTE_high_ and FLAIR_high_ yielded more prominent errors (largest absolute error was −0.11 μm^2^/ms). Overall, these simulations indicate that low errors can be obtained using either STE_high_ or FLAIR_low_, assuming a well‐matched inversion pulse for the latter approach.

#### 4.1.2 Part 2

**FIGURE 2:**
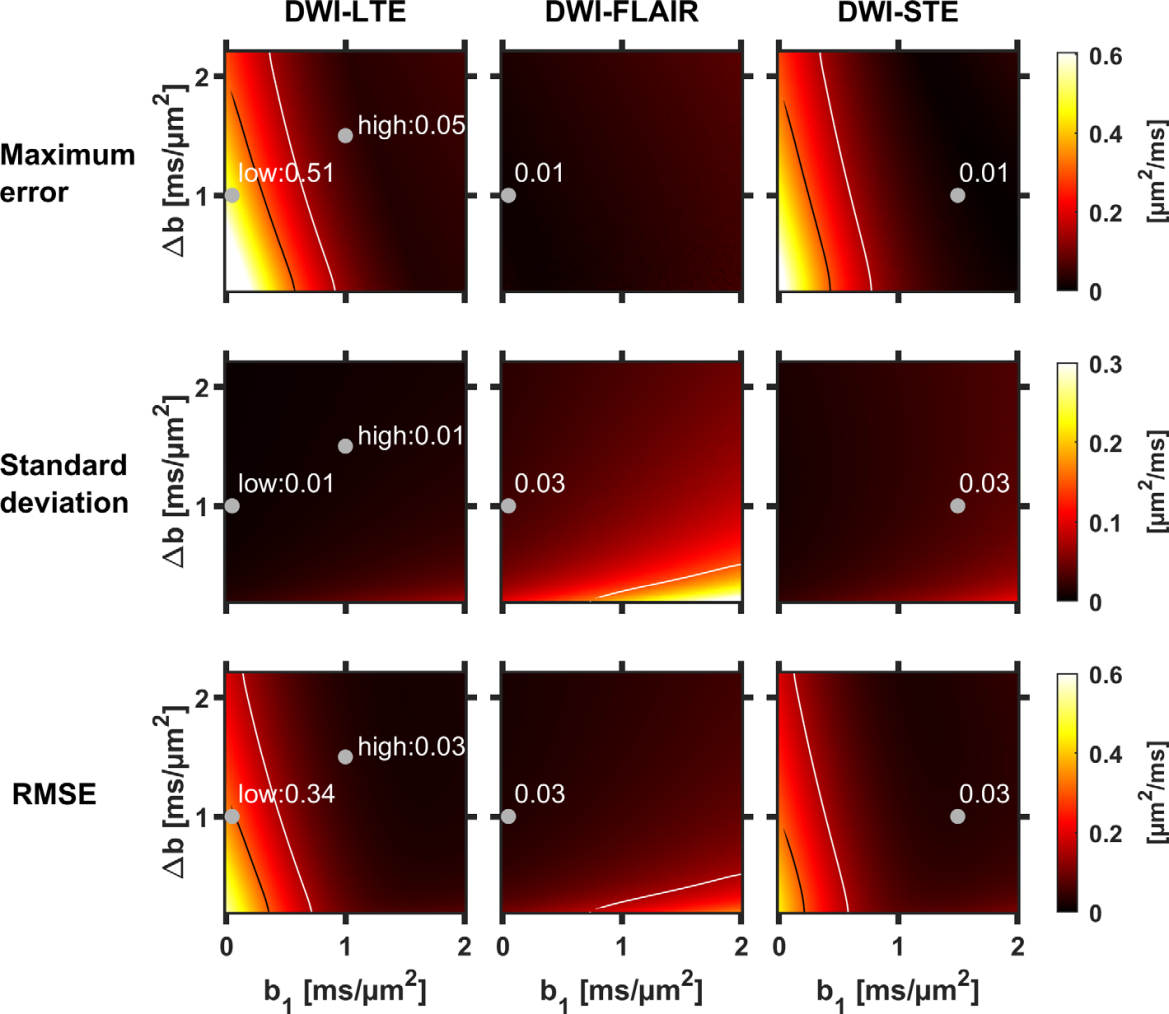
Analysis of accuracy and precision of MD in GM. Maximum errors compared to a noncontaminated signal (top row), SD over noise iterations (middle row), and RMSE compared to noncontaminated signal (bottom row) for DWI‐LTE, DWI‐FLAIR, and DWI‐STE. Points corresponding to the *b*‐value combinations used in vivo are shown in gray, and isolines marking 0.15 μm^2^/ms (white) and 0.3 μm^2^/ms (black) are included. Low *b*‐values yielded large errors for DWI‐LTE and DWI‐STE but not for DWI‐FLAIR. However, SDs were larger for DWI‐FLAIR than for the other techniques. RMSE, root‐mean‐square error.

Figure 2 shows the maximum error over the simulated variations (partial volume fractions, T_1_‐variations), SD between noise realizations, and RMSE over noise realizations and physiological variations. For both DWI‐LTE and DWI‐STE, the largest errors were obtained for low *b*‐values (*b*_1_ < 0.2 ms/μm^2^ and Δ*b* < 0.9 ms/μm^2^)—between approximately 0.4 and 0.8 μm^2^/ms for DWI‐LTE and 0.3 and 0.6 μm^2^/ms for DWI‐STE. In the high *b*‐value range (*b*_1_ > 1.3 ms/μm^2^ and Δ*b* > 0.5 ms/μm^2^), the errors were generally below 0.05 μm^2^/ms for DWI‐LTE and 0.03 μm^2^/ms for DWI‐STE. For DWI‐FLAIR, the errors were generally below 0.05 μm^2^/ms. Overall, larger errors were seen for DWI‐LTE than for DWI‐STE.

The SDs showed a similar pattern in all three cases, with low precision (high SD) at high *b*‐values, especially when combined with a small Δb. Overall, DWI‐FLAIR yielded worse precision than the other two methods because it demanded longer TR and thus allowed for fewer averages in the allotted time. Still, SDs were similar for DWI‐FLAIR and DWI‐STE for the *b*‐value combinations used in vivo.

In the RMSE maps, high values were found where CSF contamination caused poor accuracy at lower *b*‐value combinations for DWI‐LTE and DWI‐STE, as well as in DWI‐FLAIR due to poor precision.

Gray points in Figure 2 mark the *b*‐value combinations used for the in vivo data protocols. Here, the maximum errors were higher for LTE_low_ (0.51 μm^2^/ms) and LTE_high_ (0.05 μm^2^/ms) than for FLAIR_low_, (0.01 μm^2^/ms), and STE_high_ (0.01 μm^2^/ms). SDs were lower for LTE_low_ and LTE_high_ (0.01 μm^2^/ms for both) than for FLAIR_low_ (0.03 μm^2^/ms) and STE_high_ (0.03 μm^2^/ms). Relatively low RMSE were found at both the points corresponding to FLAIR_low_ (0.03 μm^2^/ms), STE_high_ (0.03 μm^2^/ms), and LTE_high_ (0.03μm^2^/ms), whereas the values for LTE_low_ were higher (0.34 μm^2^/ms). In summary, the approaches showing the lowest errors and highest precision were STE_high_ and FLAIR_low_, with LTE_high_ being a strong contender.

#### 4.1.3 Part 3

**FIGURE 3:**
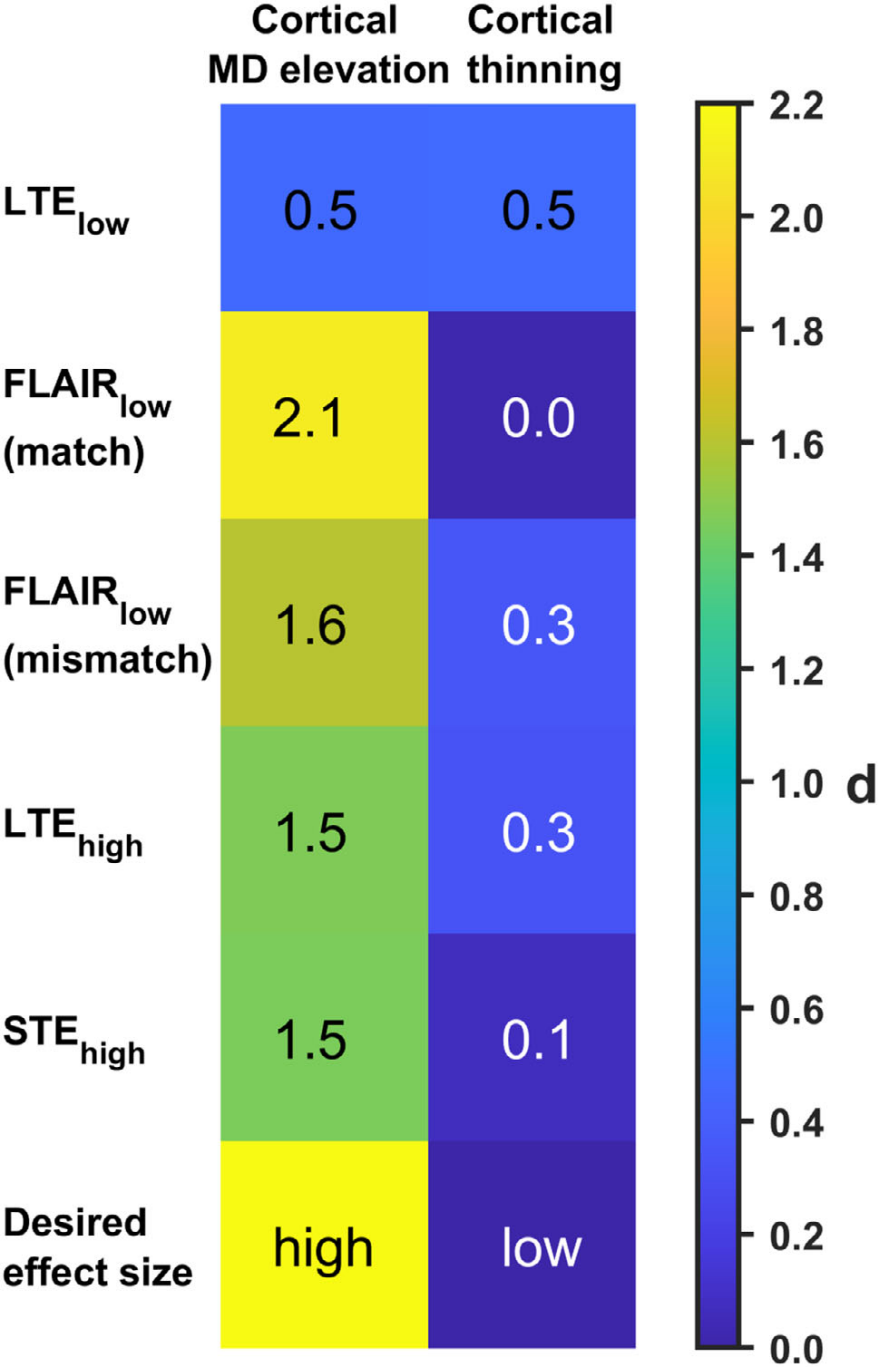
Schematic analysis of effect size. Effect sizes, in terms of Cohen's *d* for two types of GM abnormalities: global MD elevation, and cortical thinning for LTE_low_, FLAIR_low_ with perfect inversion pulse, FLAIR_low_ with inversion pulse mismatch, LTE_high_, and STE_high_. FLAIR_low_ and STE_high_ were able to distinguish the two abnormalities well. A mismatch for the inversion pulse reduced this ability. FWE, free‐water elimination.

Figure 3 shows the simulated effect sizes for two types of GM abnormalities: elevated MD in the cortex and cortical thinning with preserved cortical MD. LTE_low_ yielded similar effect sizes for both the MD elevation and cortical thinning (0.5 for both). FLAIR_low_ with perfectly timed inversion pulses yielded a large effect size for the MD elevation (2.1) and an effect size of zero for cortical thinning, but imperfect timing reduced it for the MD elevation (1.6) and increased it for cortical thinning (0.3). LTE_high_ yielded effect sizes of 1.4 and 0.3, respectively. With STE_high_, a high effect size was obtained for MD elevation (1.6) but not for cortical thinning (0.1). In summary, all of the nonconventional approaches (FLAIR_low_, LTE_high_, STE_high_) yielded the desired outcome of increasing sensitivity to a global elevation in MD while reducing the sensitivity to cortical thinning.

#### 4.1.4 In vivo

**FIGURE 4:**
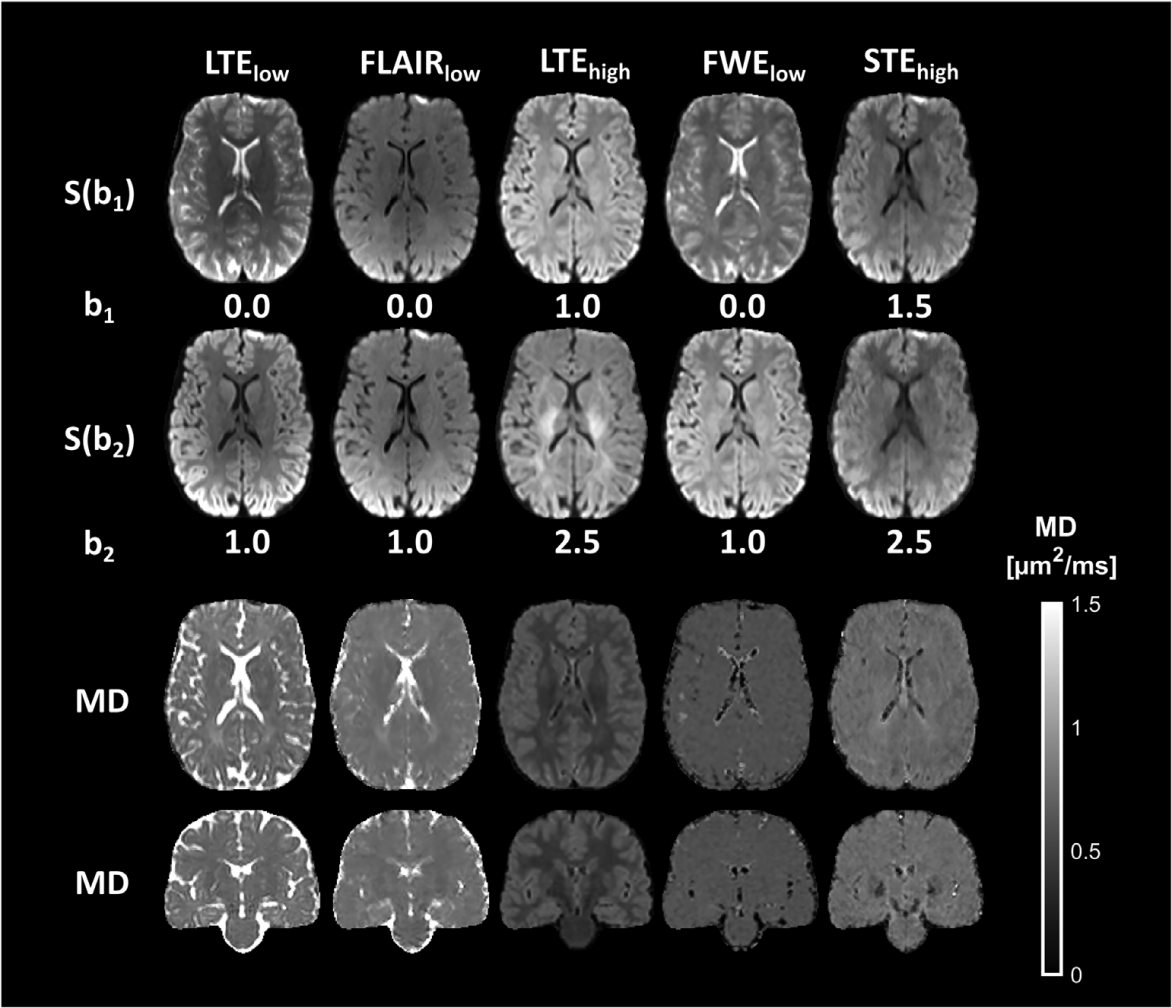
Signal intensities and MD maps from a healthy participant following coregistration to the T_1_‐MPRAGE image. First and second row: diffusion weighted images obtained with b_1_ (first row) and b_2_ (second row). Third and fourth row: transversal (third row) and coronal (fourth row) slices of MD maps obtained with LTE_low_, FLAIR_low_, LTE_high_, FWE_low_, and STE_high_. Compared to LTE_low_, CSF contributions were reduced in all cases.

Figure 4 shows transversal diffusion‐weighted images acquired with the low and high *b*‐values (*b*_1_ and *b*_2_) used to compute MD, in Equation (3), along with transversal and coronal slices of the corresponding MD maps.

All nonconventional approaches reduced the amount of PVE from CSF compared with conventional DWI (LTE_low_); however, the MD maps displayed clear differences. For FLAIR_low_, CSF contamination was reduced, but effects of residual CSF signal were observed as elevated MD close to the ventricles and the subarachnoid space. In LTE_high_, a pronounced difference in MD was seen between WM and GM due to the higher diffusional kurtosis in WM. FWE_low_ and STE_high_ both produced maps of MD without prominent CSF contamination or differences between WM and GM. A global shift in MD values could, however, be seen where FWE_low_ yielded MD values substantially below those from both STE_high_ and FLAIR_low_ (a median MD of approximately 0.55 μm^2^/ms compared with 0.7 μm^2^/ms and 0.7 μm^2^/ms, respectively).

**FIGURE 5:**
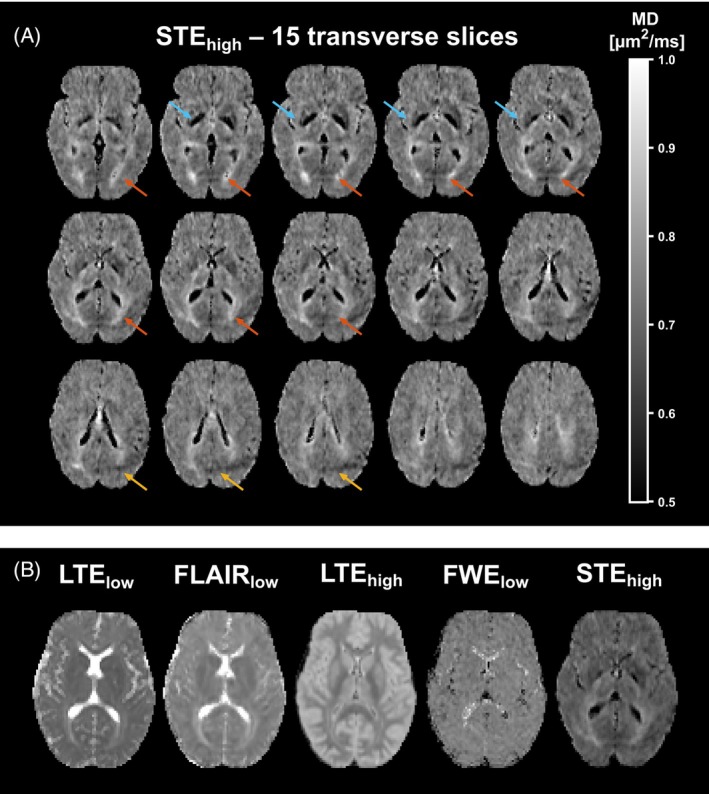
Examples of features of interest in MD in one healthy participant following coregistration to the T_1_‐MPRAGE image. (A) Fifteen transverse slices of an MD map obtained with STE_high_ in a healthy participant (23 years). The patterns identified were: diffuse hyperintensities adjacent to the posterior part of the ventricles (orange arrows), fat artifacts (yellow arrows), and hypointensities in the basal ganglia (blue arrows). (B) Transverse slices covering the optic radiation in MD maps from LTE_low_, FLAIR_low_, LTE_high_, FWE_low_, and STE_high_, where all approaches except FWE_low_ yielded hyperintense areas covering these. Image data from one subject is presented, but similar patterns were seen in all subjects studied.

In MD maps obtained with STE_high_, hyperintensities were seen posterior to the lateral ventricles and within the optic tract (orange arrows in Figure 5 and outlined in Figure S2). Elevated MD values were also seen in this region with LTE_low_, FLAIR_low_, and LTE_high_, but critically, not with FWE_low_, presumably due to the regularization used in FWE. This pattern was observed in all subjects. The MD maps obtained with STE_high_ also displayed two types of artifacts. First, hypointense regions were seen in the basal ganglia (blue arrows in Figure 5), presumably due to insufficient SNR in these regions. These hypointensities were not present in the other techniques. Second, fat artifacts were observed in the posterior parts of the brain (yellow arrows in Figure 5).

**FIGURE 6:**
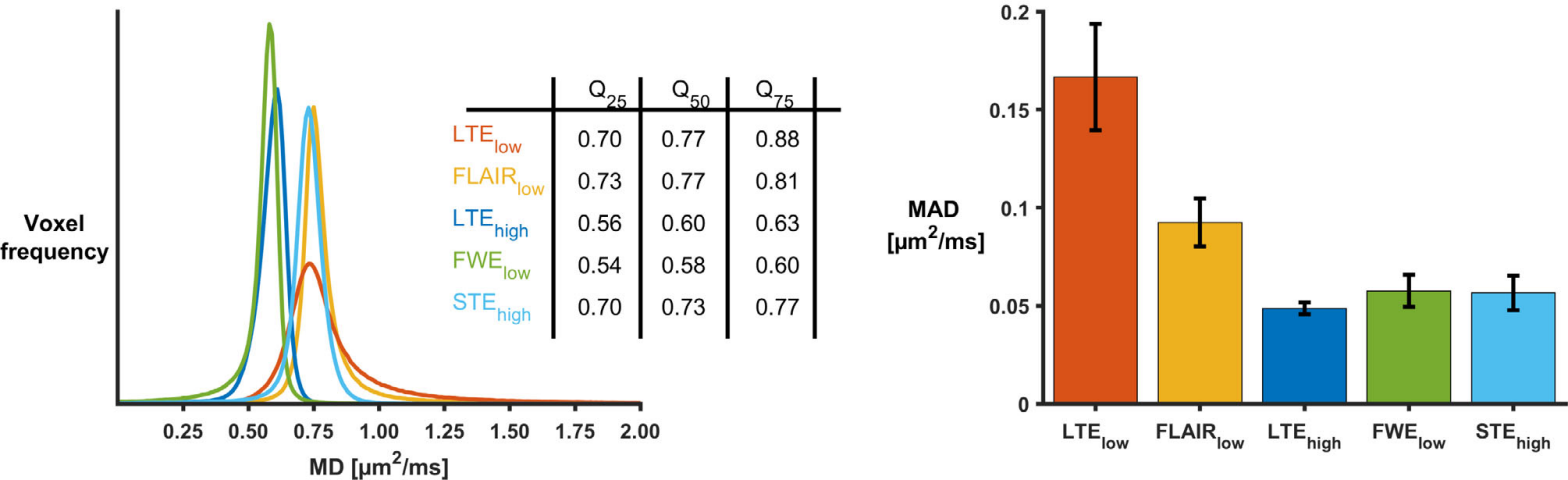
Quantitative analysis of MD values in the cortex of healthy participants. Left: histograms showing the distribution of MD values in the cortex for LTE_low_ (orange), FLAIR_low_ (yellow), LTE_high_ (dark blue), FWE_low_ (green), and STE_high_ (light blue). The 25th, 50th, and 75th quantiles of the MD values are presented for each technique. Right: bar graph showing the mean MAD of the cortical MD values in the healthy participants. All approaches reduced the number of high MD voxels and MAD of the MD values compared to LTE_low_.

The distribution of MD values in the cortical ribbon differed between the approaches (see Figure 6). Compared with LTE_low_, all nonconventional approaches had fewer voxels with MD values above 1.0 μm^2^/ms. Furthermore, STE_high_ yielded slightly lower values than FLAIR_low_, with a less pronounced tail above 1 μm^2^/ms. Lastly, both LTE_high_ and FWE_low_ yielded considerably lower values than the other techniques. The median MD values in the cortical ribbon obtained were: 0.77, 0.77, 0.60, 0.58, and 0.73 μm^2^/ms, with MADs of 0.17, 0.10, 0.05, 0.06, and 0.06 μm^2^/ms for LTE_low_, FLAIR_low_, LTE_high_, FWE_low_, and STE_high_, respectively (Figure 6). Presented in the same order, the MD values in WM were 0.76, 0.74, 0.46, 0.59, and 0.72 μm^2^/ms with MADs of 0.10, 0.05, 0.06, 0.03, and 0.04 μm^2^/ms. Notably, MADs were generally higher in GM than WM, especially for LTE_low_ and FLAIR_low_.

## 5 DISCUSSION

This study characterized five approaches for bias reduction in the mapping of cortical MD. Simulation and in vivo results showed that the largest bias in MD came from PVE with CSF in conventional dMRI using LTE_low_ (Figures 2, 4, and 6). Conventional dMRI also showed limited ability to distinguish elevated cortical MD from cortical thinning in simulations (Figure 3). The influence of CSF was reduced with FLAIR_low_, in both the simulations (Figure 2) and the in vivo data (Figures 4 and 6), although residual influence of CSF was seen near the ventricles and the subarachnoid space (Figure 4). FLAIR_low_ also displayed a low precision because the long TRs demanded by the inversion pulse limited the number of averages per unit time (Figure 2). Nonetheless, FLAIR_low_ yielded a clear separation between elevated cortical MD and cortical thinning, although the performance depended on the inversion pulse timing (Figure 3). LTE_high_ also reduced the influence of CSF, but the high *b*‐values used caused a pronounced difference in MD between GM and WM (Figure 4) and a clear reduction in MD (Figure 6). These effects are attributable to higher‐order terms and the higher kurtosis in WM (MK ≈ 1.3) than in GM (MK ≈ 0.8).^65^ STE_high_ yielded maps with suppressed CSF, with similar MD in GM and WM, and a cortical MD only slightly lower than that obtained with LTE_low_ and FLAIR_low_. Furthermore, it featured a relatively high precision and a clear ability to separate elevated cortical MD from cortical thinning (Figure 3). Finally, FWE_low_ produced flat MD maps with no discernable CSF influence, and similar MD in GM and WM (Figure 4) but a general reduction in cortical MD of approximately 0.15 μm^2^/ms compared with FLAIR_low_ and STE_high_. The approach also obscured anatomical patterns seen in the other maps, such as the elevated MD in the optic tract (Figure 5). We interpret this as a false negative result for which the approach obscures a true difference in microstructure. Although we did not investigate the cause of this effect, we note that the assumptions of the approach have been criticized^35^, ^38^ and that incorrect assumptions may introduce bias and obscure true effects.^41^ Overall, STE_high_ and FLAIR_low_ stood out as the most promising, displaying high accuracy and sufficient precision. These approaches will be discussed in more detail below.

The MD maps obtained using STE_high_ showed similar values in WM and GM (Figure 4), as expected from studies using carefully placed regions of interest or signal suppression to reduce PVE with CSF and WM.^40^, ^54^ Notably, MD values of healthy tissue obtained with STE_high_ were found within a narrow range. This allows narrow windowing when displaying the maps, which can enhance detection of abnormalities. In the presence of larger variations such as those associated with PVE with free water in LTE_low_ or WM in LTE_high_, a narrow window setting results in over‐ or undersaturation of parts of the map, which hampers its readability.

In the healthy volunteers, we found diffuse hyperintensities posterior to the ventricles and within the optic radiation (Figure 5), consistent with patterns reported for FLAIR in healthy subjects.^66^, ^67^ The cause of these patterns is unknown, but the optic tract has an unusual microstructure with an increased water content as observed by multi‐echo relaxometry,^68^ and a low fiber density as shown through staining^69^ and tractography.^70^ All approaches except FWE_low_ indicated an elevated MD here, which suggests that this reflects true differences in the microstructure rather than an artifact. However, STE_high_ did display some artifacts, for example, in the putamen and the globus pallidus, which were hypointense in the MD. This is likely a noise floor–related bias caused by low SNR due to short T_2_ relaxation times in these structures,^71^ which may stem from high iron concentrations.^72^, ^73^ Additionally, insufficient fat suppression was present, as seen as a posterior dark band (Figure 5). We have no clear explanation for the prevalence of this artifact in STE_high_ compared to the other methods. However, the slow decay of the fat signal may make this artifact more prominent at the lower SNR conditions in which STE_high_ operates. Furthermore, the waveform used in STE_high_ is applied repeatedly throughout the whole image acquisition, which may cause long‐term buildup of eddy currents. Methods to reduce fat artifacts have been suggested^74^ and will be investigated in the future.

The high *b*‐values used in STE_high_ reduced PVE with CSF, which reduced the maximal error in the simulations from 0.51 to 0.01 μm^2^/ms with LTE_low_ and STE_high_, respectively (Figure 2). It also allowed a more distinct separation between cortical MD elevation and cortical thinning (Figure 3). This aligns with previous research, where increased MD accuracy was obtained by increasing minimum *b*‐values (from 0 to 0.5 ms/μm^2^ or 0.6 ms/μm^2^).^33^, ^75^ To our knowledge, no study has estimated MD values with minimal *b*‐value as high as 1.5 ms/μm^2^, where only 1% of the CSF signal remains compared to 22% at 0.5 ms/μm^2^. This may be because higher *b*‐values are associated with increased bias from higher‐order terms such as the diffusional kurtosis. In uncontaminated GM voxels, biases of approximately −0.08 μm^2^/ms and −0.3 μm^2^/ms are expected at LTE_low_ and LTE_high_, respectively (Figure 1). This error is reduced to approximately −0.03 and −0.15 μm^2^/ms for STE_low_ and STE_high_, respectively, because STE does not encode the anisotropic part of the kurtosis. A bias of −0.15 μm^2^/ms is not negligible but has to be compared with that caused by PVE with CSF, where a signal contamination of merely 10% is expected to yield a bias of approximately 0.15 μm^2^/ms in conventional MD mapping with LTE_low_ (Figure 1). Furthermore, the kurtosis‐related bias is expected to be relatively constant throughout the cortex. For this reason, errors in the simulations were measured relative to the MD in an uncontaminated voxel rather than to the ground truth value.

The benefits of STE come at a cost. First, STE thrives on strong gradients.^47^ This study utilized a system with a maximum gradient amplitude of 80 mT/m. Systems with weaker gradients can use STE but at longer TEs and therefore reduced SNR or poorer image resolution.^47^ Second, STE demands longer TEs than LTE for the same *b*‐value, which further reduces SNR. In our study, a TE of 110 ms was used, compared to TEs of 60–80 ms for the LTE sequences, corresponding to an SNR reduction of 30%–40% in GM. However, these effects were included in the simulations, which did not indicate precision to be a major problem. Additionally, STE causes a stronger signal attenuation than LTE, which, alongside high *b*‐value encoding, may yield bias from the rectified noise floor. As in this study, this can be mediated through super‐resolution reconstruction,^42^, ^76^ for example, by acquiring thick slices combined with slab rotation.

The second approach that gave promising results for cortical MD mapping was FLAIR_low_. Here, the simulations indicated improved MD accuracy, with errors generally below 0.05 μm^2^/ms (Figure 2), and effect sizes that were high for cortical MD elevations and low for cortical thinning (Figure 3). These results were obtained despite the inclusion of fluctuations in T_1_ of CSF caused by variations in body temperature. Thus, normal fluctuations in body temperature are not expected to cause major detrimental effects on MD accuracy. Still, T_1_‐values of CSF reported in earlier studies (3.8–6.9 s)^53^ span a much larger range than that stemming from realistic in vivo temperature variations (4.5–4.6 s). Thus, individual variations in T_1_ of CSF may be more extensive than those included in the simulations. Furthermore, the in vivo results differed substantially from the simulation results as imperfect fluid suppression was seen in all subjects (Figure 4). This also manifested as a tail in the distribution of cortical MD values above 1.0 μm^2^/ms (Figure 5), and a larger MAD of FLAIR_low_ compared with LTE_high_, FWE_low_, and STE_high_. With the current protocol setup, an unexpectedly short TI was needed to achieve sufficient fluid suppression, that is, about 0.5 s shorter than calculated from Equation 10, assuming T_1_ = 4.5 s and TR and TE are taken from the protocol. Further investigation revealed that the optimal TI depended on the slice gap, where larger slice gaps brought TI closer to its theoretical value (Figure S3). This results in a dilemma when using DWI‐FLAIR: One has to accept substantial slice gaps or imperfect fluid suppression. Another shortfall of FLAIR_low_ is its low precision (Figure 2) due to the long TR required for the inversion, even when SRR was used to mitigate this. In simulations, the precision with FLAIR_low_ was nonetheless comparable to that of STE_high_, but it was slightly worse in vivo due to residual PVE with CSF.

### 5.1 Limitations and future work

A limitation of the current study is the variability of the protocols. This includes a variation in the multiband acceleration factor, partial Fourier factor, and total acquisition time (see Table 2). First, SMS was not used for LTE_low_ due to an omission in the protocol setup. Using SMS would have improved the precision of LTE_low_ by allowing additional repetitions; however, the precision was not the limiting factor for this approach, as seen in the simulation (Figure 2). Second, different partial Fourier factors were used in LTE_high_ and FWE_low_, again, due to an oversight in the protocol setup. Although matching partial Fourier factors would have been preferred, we expect the impact of this variation to be relatively small. Third, variation in the acquisition times hampers a direct comparison between some of the approaches in terms of use and application, but again, precision was not generally the limiting factor for the various approaches.

The protocols used in this study can be further optimized before they are applied in future research. Nonetheless, we expect the trends and mechanisms described in this work to remain for optimized protocols. Additionally, further investigation is needed to assess the benefits of increased image resolutions. For example, Little et al. obtained relatively high‐resolution images acquired within a feasible time (1.5 mm isotropic in 3:30 min) but reported results possibly affected by PVE. Even higher resolutions have demanded substantially longer scan times. Thus, the balance between reduced PVE and increased scan time needs to be investigated. Lastly, additional investigations of post‐processing approaches are warranted. This includes, among other steps, Gibbs ringing corrections because its impact on accuracy and effect sizes for cortical MD has yet to be evaluated. The FWE method used is based on the extensively used regularized gradient descent approach, but other algorithms are also available. Future work is needed to validate such algorithms using independent data, which can advantageously be done using nonconventional encoding.^77^

## 6 CONCLUSION

We investigated the strengths and limitations of established approaches for mapping cortical MD, along with a new approach utilizing spherical b‐tensor encoding at high *b*‐values to reduce bias from partial volume effects with WM and CSF, and super‐resolution acquisition and reconstruction to reduce bias from the noise floor. Results showed that high accuracy could be obtained using the proposed approach, while avoiding potential pitfalls such as imperfect CSF nulling with FLAIR and errors from model assumptions with FWE algorithms.

## Supporting information


**FIGURE S1.** The mean signal extracted from the ventricles (squares), and GM (circles), in one slice of a test subject at different inversion times. A relatively flat minimum for the free water signal is present around 1.5 s. The signal in GM increased with the inversion time.
**Figure S2.** The optic radiations (segmented in tractseg) shown over the corresponding slices of an MD map obtained from STE_high_ data.
**Figure S3.** Analysis of the free water signal at different slice gaps and inversion times. The free water signal as a function of slice gap (left) for FLAIR_low_ and STE_high_. The free water signal as a function of inversion times at different slice gaps for FLAIR_low_. Overall, the fluid suppression in FLAIR_low_ showed a clear slice gap dependency that was not present for STE_high_.
