# Supplementary material for: Diffusion MRI in the cortex of the brain: Reducing partial volume effects from CSF and white matter in the mean diffusivity using high b‐values and spherical b‐tensor encoding
Source: Magn Reson Med. 2025 Jun 4;94(3):1166–81. doi: 10.1002/mrm.30552 (PMC12202729; doi:10.1002/mrm.30552)
Supplement: Supplementary file 1 — FIGURE S1. The mean signal extracted from the ventricles (squares), and GM (circles), in one slice of a test subject at different inversion times. A relatively flat minimum for the free water signal is present around 1.5 s. The signal in GM increased with the inversion time. Figure S2. The optic radiations (segmented in tractseg) shown over the corresponding slices of an MD map obtained from STEhigh data. Figure S3. Analysis of the free water signal at different slice gaps and inversion times. The free water signal as a function of slice gap (left) for FLAIRlow and STEhigh. The free water signal as a function of inversion times at different slice gaps for FLAIRlow. Overall, the fluid suppression in FLAIRlow showed a clear slice gap dependency that was not present for STEhigh. [file MRM-94-1166-s001.docx]

# Supporting information

#
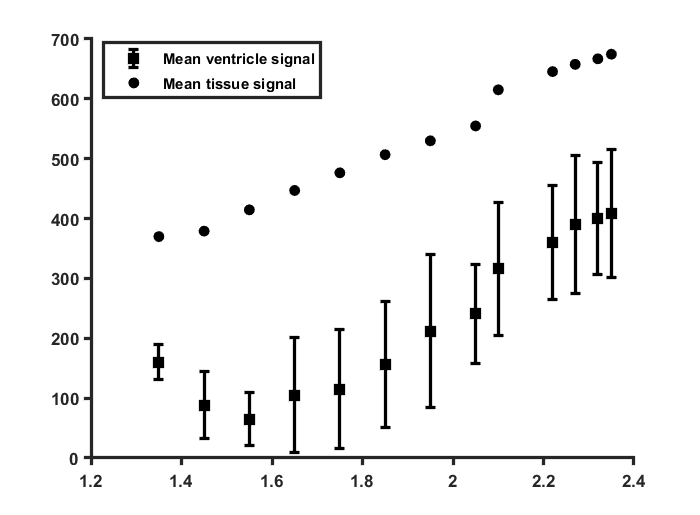


Figure S1: The mean signal extracted from the ventricles (squares), and GM (circles), in one slice of a test subject at different inversion times. A relatively flat minimum for the free water signal is present around 1.5 s. The signal in GM increased with the inversion time.


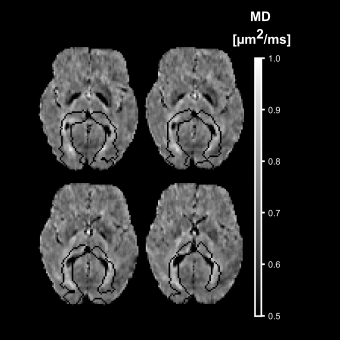


Figure S2: The optic radiations (segmented in tractseg) shown over the corresponding slices of an MD map obtained from STE_high_ data.

# Investigation of fluid suppression with FLAIR_low_ and STE_high_

### Methods

Further investigation of the effects of slice gap on fluid suppression efficiency was performed. This was done by collecting b = 0 data for FLAIR_low_ and STE_high_, with a range of slice gaps (0 –100 %), and, for FLAIR_low_, a range of Tis of (1.3 – 2.9 s). Signal values were extracted from the ventricles in one slice.

### Results

Figure S3 shows the ventricle signal with different slice gaps and inversion times. FLAIR_low_ yielded a more pronounced slice gap dependency than STE_high_. Furthermore, STE_high_ produced a more complete fluid suppression than STE_high_. The optimal TI increased with increasing slice gap from around 1.5 s at 0 % slice gap to 2.1 s at 100 % slice gap. At 100 % slice gap, a more complete fluid suppression was obtained.


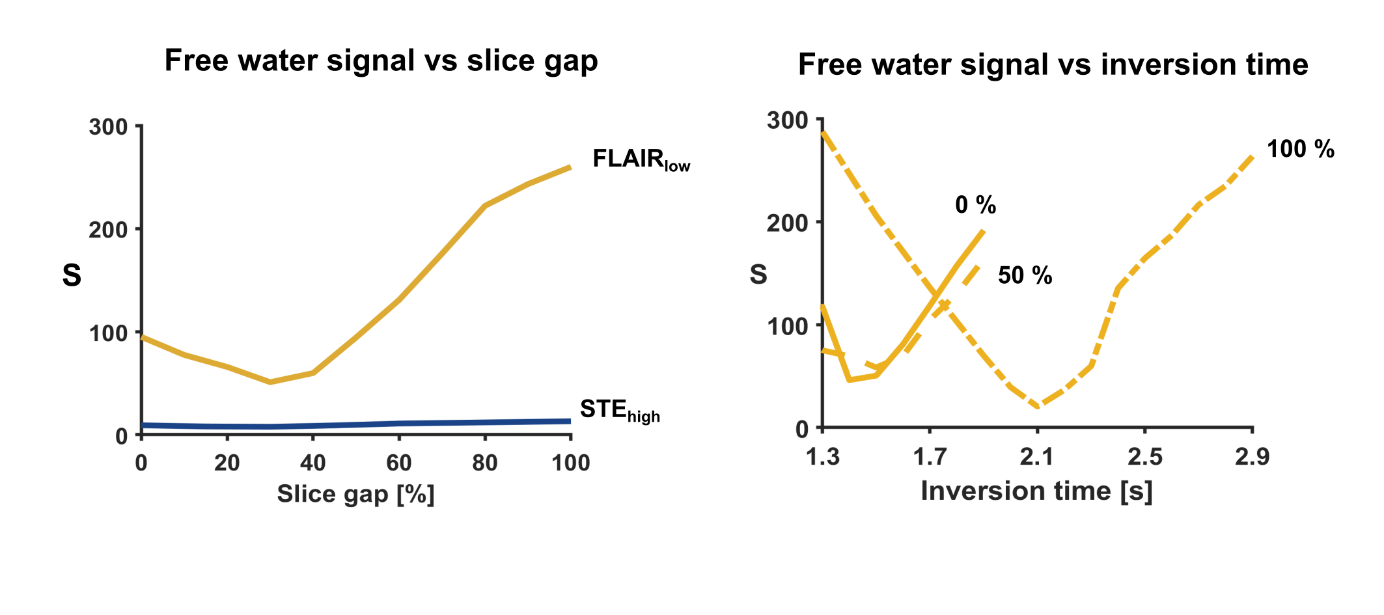


Figure S3: Analysis of the free water signal at different slice gaps and inversion times. The free water signal as a function of slice gap (left) for FLAIR_low_ and STE_high_. The free water signal as a function of inversion times at different slice gaps for FLAIR_low_. Overall, the fluid suppression in FLAIR_low_ showed a clear slice gap dependency that was not present for STE_high_.
